# Beyond Immediate Individual Care: Monitoring Captured Free-Ranging European Wild Ungulates to Refine Protocols

**DOI:** 10.3390/vetsci13070705

**Published:** 2026-07-18

**Authors:** Jorge Ramón López-Olvera

**Affiliations:** Wildlife Ecology & Health Group (WE&H) and Servei d’Ecopatologia de Fauna Salvatge (SEFaS), Departament de Medicina i Cirurgia Animals, Facultat de Veterinària, Universitat Autònoma de Barcelona (UAB), Bellaterra, 08193 Barcelona, Spain; elrebeco@yahoo.es; Tel.: +34-650110233

**Keywords:** assessment, azotaemia, capture myopathy, catecholamines, monitoring, muscular enzymes, protocol, renal vasoconstriction, stress

## Abstract

European wild ungulates are captured for different purposes, requiring the assessment of animal welfare and health. Specific guidelines to assess stress and pathophysiological compromise in captured wild ungulates are lacking. This review aims to set the bases for establishing standardised protocols allowing the evaluation of capture and handling stress in wild European ungulates. Physical and chemical capture and handling elicit stress, a physiological response activating sympathetic–adrenal medulla and hypothalamic–pituitary–adrenal cortex axes, releasing catecholamines and corticosteroids, respectively. While being an adaptive response, if prolonged over time, the effects become harmful and life-threatening, increasing body temperature and heart rate, and provoking muscular and renal ischemia. This clinical outcome is known as capture myopathy, which encompasses four sequential syndromes: Hyper acute or capture shock, Acute or ataxic-myoglobinuric, Sub-acute or ruptured muscle, and Chronic debility or delayed per-acute. Monitoring temperature, heart rate, serum muscular enzyme (CK, AST, ALT, and LDH) activities and lactate, creatinine, urea, and potassium concentrations characterises pathophysiological compromise. Wild ungulate capture and handling protocols should include not only methodology but also monitoring, data recording, sample collection, and analyses. This information should serve to improve protocols and eventually develop specific guidelines for stress and welfare assessment when capturing and handling wild ungulates.

## Simple Summary

The capture and manipulation of European wild ungulates is often necessary but causes stress and may threaten the life of the animals. Assessing stress is essential to identify the best practices and improve animal welfare, health, and survival. This review summarises the current knowledge on stress evaluation in captured European wild ungulates and proposes a monitoring methodology to improve capture and handling protocols. The physiological changes caused by capture affect body temperature, heart rate, muscle, and kidneys among other organs, with consequences for the whole body. The clinical indicators of these effects must be analysed when capturing and handling wild ungulates, not only to monitor the specific captured individual but to assess, revise, and improve the whole protocol. Ideally, the information collected should serve to establish common guidelines to capture, manipulate, and monitor wild ungulates captured in Europe.

## 1. Introduction

Wild ungulates in Europe are captured for population restocking, scientific studies (related to health, behaviour or population), to mark or translocate animals, to treat diseases, to obtain biological samples or as a management measure for overabundance [1,2,3,4,5,6,7,8]. Additionally, the relatively recent colonisation by wild ungulates (mainly wild boar) of urban and peri-urban environments in different European cities [9,10,11,12,13,14,15,16,17,18,19], where hunting is unfeasible or not allowed by law due to safety reasons, has further triggered the need for capture methods to manage these synurbic populations [1,20,21,22,23]. Capture and culling have also been extensively used as a measure aimed at local population eradication in the management of disease outbreaks such as African swine fever [22,24,25,26].

Traditionally live capture of wildlife was viewed solely as a means of achieving a specific objective, with the resulting stress and associated risks considered unavoidable issues to be managed. The studies initially focused on the suitability, performance, cost, selectivity, capability of capturing social groups, personnel needs, effort, and other features of methods to capture a given species or group of species. However, the welfare implications of wildlife capture have progressively become a field of research, driven by increasing societal concern for animal welfare [1,5,6,27,28,29,30,31,32,33,34,35,36,37,38,39,40,41,42,43,44,45].

Regardless of the objective of the capture operation, the responsibility of professionals working with animals, including wildlife, and increased social sensitivity require the assessment of the impact of capture on health and welfare, even if the final objective of the capture is the death of the animal [20,23]. Guidelines for assessing welfare of captured wildlife are available for different taxa [46,47,48,49,50,51,52]. However, (1) most of these guidelines focus on traumatic injuries and immediate capture mechanical death, ignoring stress-mediate impact on health, welfare, and survival; and (2) few if any are specifically developed for wild ungulates, despite being among the most frequently captured and handled wildlife taxa. The objective of this review is to provide a comprehensive summary of the existing knowledge on European wild ungulate capture stress assessment, setting the bases for establishing standardised protocols allowing the evaluation of the stress provoked by capture and management procedures, thus allowing improvement, refinement, and comparison among methodologies.

## 2. Capture and Handling of European Wild Ungulates

Three different approaches have been used to capture and handle wild European ungulates: physical capture, teleanaesthesia (i.e., remote chemical immobilisation using distance-delivery systems), and physical capture followed by anaesthesia. Box-traps, corral-traps, and different net types, including mainly drop-nets and drive-nets, are the physical methods most frequently used in Europe (Table 1). Medium-sized ungulates can be physically captured and handled without anaesthesia. Although few studies have directly compared the mortality and stress induced by physical capture and teleanaesthesia in free-ranging wild ungulates [35,36,53,54,55], the overall results seem to point that the risks of on-field teleanaesthesia are higher than those of physical capture methods [5,35,36,37,53,55,56]. However, the manipulation of bigger species or the realisation of long, disturbing, and/or painful procedures recommend and impose the use of anaesthesia, for both human and animal welfare and safety reasons [57]. Thus, bigger species such as European bison (*Bison bonasus*), moose (*Alces alces*), muskox (*Ovibos moschatus*), and even red (*Cervus elaphus*) and fallow (*Dama dama*) deer are usually or most frequently captured and manipulated under anaesthesia (Table 1). Teleanaesthesia has also been used to capture smaller species such as Alpine (*Capra ibex*) and Iberian (*Capra pyrenaica*) ibexes, European mouflon (*Ovis aries musimon*), and Northern (*Rupicapra rupicapra*) and Southern (*Rupicapra pyrenaica*) chamois, mainly for long, painful, or particularly stressful procedures and interventions, as well as in wild boar (*Sus scrofa*), whose manipulation can be potentially harmful for humans [58] (Table 1). Finally, the conceptually relevant difference between capture and immobilisation has led to the combined use of anaesthesia for immobilisation after physical capture in several of the abovementioned species (Table 1).

## 3. Stress Response in Wild Ungulates

Whatever the capture method, wild ungulates undergo a physiological stress response to capture and manipulation. Stress is a physiological adaptive mechanism that is continuously activated to maintain homeostasis in response to both internal and external challenges [136,137,138,139]. Physical restraint and/or immobilisation are among the stressors which elicit the strongest stress response [140]. Capture-related stress is a specific anticipatory response elicited even by human approach, so any animal captured, handled, or even in proximity of people has already an activated physiological response [141,142]. Therefore, basal values for any physiological, haematological, biochemical, or clinical variable in free-ranging wildlife cannot be established, since the recording of such information or the obtention of the samples required will be already affected by the required approach and manipulation, so specific reference values have to be established for each capture and handling protocol [77,112,121]. Developing sampling and monitoring methodologies that do not elicit a stress response is an unsolved challenge that would help to achieve precise knowledge of the stress response to capture and handling. Since this is an anticipatory response triggered even by the animal moving out of its refuge area or simple human approach [141,142], this physiological response also occurs in anaesthetized wildlife, either free-ranging or in captivity [62,143,144,145]. Captivity and repeated captures can cause either sensitization (i.e., stronger stress response to the same stimulus) or adaption (i.e., reduced stress response). Thus, even captive-kept or tame wildlife experience stress as a physiological response to capture, handling, and manipulation, although it may be modified because of sensitization or adaption [89,113,118,119,146].

**Figure 1:**
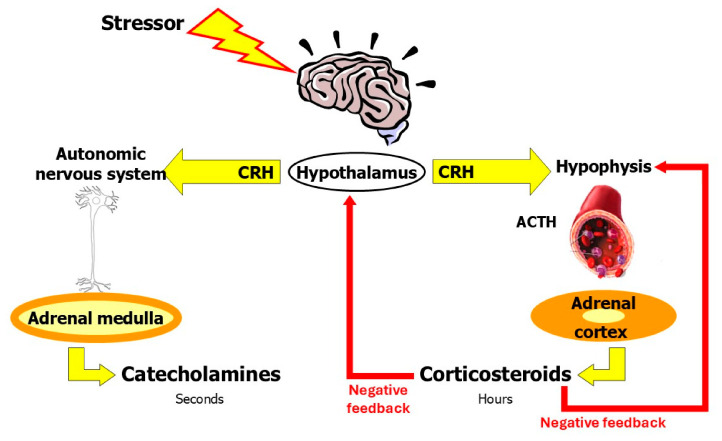
Physiological pathways triggered by stressors activating the sympathetic–adrenal medulla (SA) and the hypothalamic–pituitary–adrenal cortex (HPA) axes via corticotrophin-releasing-hormone (CRH) to release catecholamines (SA) and corticosteroids via adrenocorticotropic hormone (ACTH; HPA), respectively.

Stress response has been traditionally divided in three stages: (1) perception of an external or internal stimulus, known as stressor, as a threat to homeostasis; (2) physiological stress response *sensu stricto*, triggered by the stimulus as the activation by corticotrophin-releasing-hormone (CRH) of the sympathetic–adrenal medulla (SA) and the hypothalamic–pituitary–adrenal cortex (HPA) axes, which will release catecholamines (SA) and corticosteroids via adrenocorticotropic hormone (ACTH; HPA), respectively; and (3) biological consequences of the action of catecholamines and corticosteroids [138,140,147,148,149,150,151,152,153,154] (Figure 1).

Catecholamines and corticosteroids are involved in the regulation of physiological mechanisms in practically all the body systems [155,156,157,158,159,160,161,162]. While both the HPA and SA axes are activated by the exposure to a stressor, the release of catecholamines is immediate due to the neuronal nature of the SA response [140,163]. Corticosteroids released by the activation of the HPA axis take longer to act since they are released through the bloodstream. Corticosteroids are responsible for terminating the acute stress response and shifting to the chronic stress response through reflex parasympathetic activation and negative feedback inhibition. These mechanisms stop the release of CRH and ACTH from hypothalamus and hypophysis, respectively, thus regulating not only corticosteroids but also catecholamines [140,164] (Figure 1).

While being an adaptive response, thus necessary to maintain homeostasis and survive as well as to respond to threats and challenges, when the stressor persists over time and the animal cannot escape from the perceived threat, the prolongation of the action of catecholamines and corticosteroids becomes harmful and life-threatening. This allows the differentiation of (1) eustress, defined as the physiological responses enabling the organism to cope with stressful situations; (2) neutral stress, which is neither harmful not helpful; and (3) distress, involving potential pathological changes [136,139,165].

**Figure 2:**
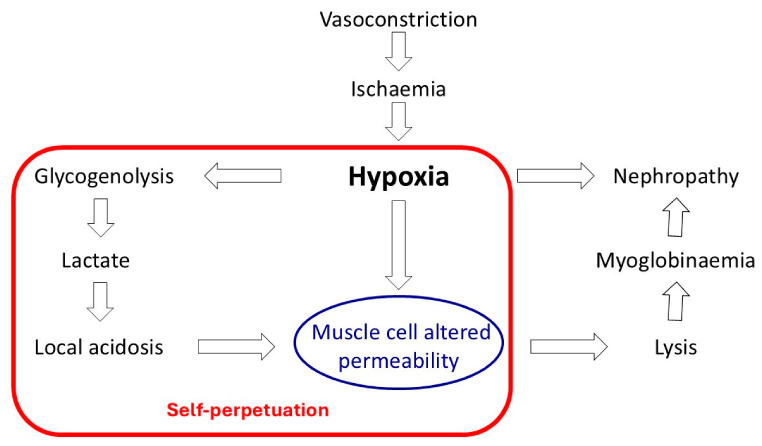
Pathophysiological mechanisms participating in capture myopathy.

Catecholamines increase body temperature and heart rate, and catecholamine-induced vasoconstriction leads to local ischemia, with effects on both muscles and kidneys. In the muscle, ischemia progressively provokes cell hypoxia, local acidosis because of the generation of lactic acid from anaerobic glycogenolysis, increased cell permeability, and eventually cell death [138,166,167,168]. The persistence of these factors due to the reduced blood perfusion leads to a self-perpetuating cycle. The myoglobin released to the bloodstream from the injured muscle cells causes nephrotoxicity, adding to the kidney compromise already posed by vasoconstriction, and leading eventually to acute kidney injury [169,170,171,172,173,174,175,176,177,178] (Figure 2).

The clinical outcome of these pathological changes in restrained wildlife is known as capture or exertional myopathy, whose pathophysiological mechanisms and consequences have been extensively and deeply described in detail. Capture myopathy occurs through four syndromes that can appear in sequential order, namely: (1) Hyper acute or capture shock; (2) Acute or ataxic-myoglobinuric; (3) Sub-acute or ruptured muscle; and (4) Chronic debility or delayed per-acute [170,171,174,175,176,177]. These syndromes are nothing but a classification into stages of the continuous pathophysiological process triggered by the stressor and mediated by catecholamines and corticosteroids if maintained over time, and death can occur at any of these stages.

**Figure 3:**
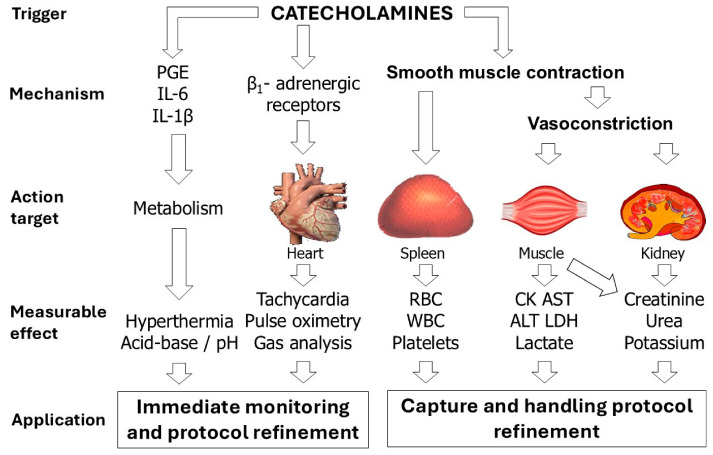
Main catecholamine-induced pathophysiological changes, measurable effects, and applicability for immediate monitoring and protocol refinement of such changes. ALT: alanine aminotransferase; AST: aspartate aminotransferase; CK: creatine kinase; LDH: lactate dehydrogenase; IL-1: interleukin 1; IL-6: interleukin 6; PGE: prostaglandin E; RBC: erythrocytes; WBC: leucocytes.

The pathophysiological consequences of stress driving to capture myopathy originate mostly from the effects of catecholamines on the metabolism and heart, as well as from the consequences of catecholamine-induced smooth muscle vasoconstriction in muscle and kidney (Figure 3).

Catecholamines are responsible for stress-induced hyperthermia (SIH), a 1.0 to 1.5 °C increase in temperature mediated by prostaglandin E and interleukins 1β and 6 [136,137,179,180,181,182,183,184] (Figure 3). This response occurs both in physically captured and anaesthetized animals and is not related to physical activity or environmental temperature [144,145], although these two factors may act synergically and worsen the clinical outcome and prognosis [171].

Catecholamine stimulation of β_1_-adrenergic receptors causes an increase in heart contractibility and heart rate [180] (Figure 3), which is considered an objective reliable indicator to assess the autonomic nervous system response to stressors [136,185]. The action of catecholamines on α-adrenergic receptors induces smooth muscle contraction in the spleen capsule, releasing erythrocytes and platelets to the bloodstream. This smooth muscle contraction also provokes the mobilisation into the circulation of leucocytes from leucocyte marginal pools, such as capillary beds and lymph nodes. This causes an increase in erythrocyte count, haemoglobin concentration, and haematocrit, as well as the catecholamine-induced phase of the so-called stress leucogram, consisting in leucocytosis with neutrophilia and lymphocytosis [137,138,186,187,188,189,190,191,192,193,194,195] (Figure 3).

In the striated muscle, the hypoxia and anaerobic glycogenolysis consequent to the vasoconstriction caused by the catecholamine-induced smooth muscle contraction increase serum lactate concentration, leading to acidosis [137,176,196,197,198,199,200] (Figure 1). The progression of the pathophysiological process leads to increased muscle cell membrane permeability, provoking leakage of muscular cell contents to the bloodstream. This can be detected by a rise in serum creatine kinase (CK), aspartate aminotransferase (AST), alanine aminotransferase (ALT), and lactate dehydrogenase (LDH) activities, as well as serum creatinine, urea, and potassium concentrations [136,169,172,173,178,188,201] (Figure 3). Renal vasoconstriction and myoglobin toxicity impair kidney filtration and reduce blood metabolite clearance, further contributing to the increase in serum creatinine, urea, and potassium concentrations [138,169,172,173,176,178,202,203,204,205] (Figure 3).

To summarise, these pathophysiological mechanisms act in each of the four syndromes of capture myopathy as follows [170,171,174,175,176,177]:

- Hyper acute or capture shock syndrome: the tachycardia induced by catecholamines leads to heart fibrillation and cardiac arrest. This syndrome is the first one that can occur, usually within six hours from the triggering of the stress response. Since this is a functional shock, little if any pathologic findings can be detected in *post-mortem* examination.
- Acute or ataxic myoglobinuric syndrome: if the animals survives to the initial shock, leakage of muscular cell contents because of increased permeability due to hypoxia, acidosis, and increased temperature increase the blood activities of muscular enzymes and concentrations of creatinine, urea, potassium and myoglobin. Renal vasoconstriction also induces hypoxia and acidosis in the kidney. When the metabolites leaked from the muscular tissue reach the kidney, they increase kidney cell toxicity and can be detected in urine. Clinically, the affected animals appear ataxic because of muscular cell rupture and the effects of uraemia on the central nervous system, and myoglobinuria gives urine a chocolate brown to blackish colour. This is the most frequent of the four syndromes, occurring usually within hours to days from the stressor.
- Sub-acute or ruptured muscle syndrome: a stressed animal that has suffered from muscular hypoxia and acidosis not intense enough to cause death may still experience muscular rupture afterwards because of cell necrosis. In ungulates, hindlimb muscles are the most frequently affected, since they have the bigger muscular masses where blood supply can be more dramatically affected. Clinical signs such as hindlimb flaccidity and tarsal hyperflexion may appear in ungulates as soon as 24 to 48 h after the stressor, usually self-perpetuating and leading to death within days or weeks.
- Chronic debility or delayed per-acute syndrome: this rare last syndrome occurs in animals that have been exposed to two episodes of intense stress separated by an interval of at least 24 h and up to approximately two weeks. Although the animal survives the first stress episode without succumbing to shock, ataxia–myoglobinuria, or ruptured muscle (i.e., the first three syndromes in chronological order), the potassium released from damaged muscle cells during the initial episode results in persistent hyperkalaemia. Since potassium participates in heart contraction, hyperkalaemia sensitises cardiac muscle to the action of catecholamines, so the chances for per-acute heart fibrillation in a second stress episode while blood potassium concentration is still elevated are higher, causing immediate death almost instantaneously.

## 4. Effect of Anaesthesia on the Pathophysiology of Capture Stress

Anaesthetizing free-ranging wildlife is probably the most challenging veterinary anaesthesia intervention, since there is a complete lack of knowledge of the pre-anaesthetic status of the individual and usually the maximum available information is species, sex and, if lucky, approximate weight, so the uncontrolled variables and probabilities of complications to appear are disproportionately higher as compared to domestic animals [57,120,206,207,208,209,210]. Moreover, the pathophysiological processes of capture stress also keep occurring even in anaesthetized wildlife [62,143,144,145]. Therefore, anaesthetized wild animals are simultaneously subjected to the physiological effects of capture-induced stress and anaesthesia, which may interact synergistically, antagonistically, or through partial overlap. Such physiological challenge is even higher when the anaesthetized individual has been either actively chased for a long time and/or distance before the anaesthesia, or when anaesthesia follows physical capture, since in both cases the stress response has been already triggered and established before administering the anaesthesia [211].

As compared to the physiological effects of capture stress, most tranquilising and anaesthetic protocols induce hypotension due to vasodilation [61,64,71,72,78,79,80,81,86,87,88,89,111,120,122,123,124,206,207,208,212,213,214,215]. This vasodilation effect could balance catecholamine-induced vasoconstriction, improving muscle and kidney blood flow and reducing the pathophysiological compromise related to capture stress [78,79,80,81,86,87,88,89,120,122,123,124]. However, anaesthetic hypotension is usually also associated with hypoxaemia due to vasodilation, haemodilution, and splenic sequestration of erythrocytes [54,55,111,120,187,190,194,211,215,216,217], which could act synergistically with the tissular hypoxia generated by catecholamines, therefore increasing muscular acidosis and consequently cell rupture and kidney toxicity.

Altogether, the interaction of the pathophysiological effects of capture stress and wildlife anaesthesia introduces significant variability and uncertainty in the welfare, health, and surviving outcome.

## 5. Measuring and Monitoring Stress in Captured Wild Ungulates

The pathophysiological changes in capture myopathy are reflected in clinical, haematological, and serum biochemical variables that can be assessed and monitored (Figure 3). Thus, temperature is a reliable indicator of SIH, and heart rate informs about catecholamine-induced tachycardia, thus assessing the activation of the autonomic nervous system. Therefore, temperature and heart are related to the intensity of the alarm perceived by the animal [136,184,185]. Measuring pH and investigating acid-base balance can indicate the acidosis status of the individual, although concerns and limitations about on-field method accuracy recommend combining the analysis of this variable with other indicators, such as lactate [71,171,203,218,219]. Similarly, on-field assessment of oxygen saturation, e.g., using pulse oximetry, and gas analysis allow the immediate monitoring of hypoxia, particularly in anaesthetized animals [61,64,71,120,220].

Blood cell count reflects catecholamine-induced smooth muscle contraction in the spleen [54,55,77,78,79,80,86,87,88,89,112,121,122,123,124,187,215]. Differential leucocyte count shift from catecholamine-induced lymphocytosis to corticosteroid-dependent neutrophilia can be useful to monitor the time the stress response has been running, since the corticosteroid effects on leucocyte differential count take longer than those of catecholamines and are usually visible only after four hours at least [188,190,193].

The most welfare-, health-, and life-threatening effects of catecholamines in capture-related stress on muscle and kidney can also be assessed through serum CK, AST, ALT, and LDH activities and lactate, creatinine, urea and potassium concentrations [54,62,77,78,79,86,87,88,89,112,121,122,123,124] (Figure 3). Serum enzymatic activities increase exponentially with the severity of muscle damage and are therefore valuable prognostic indicators [177,186,201,221,222]. CK is very sensitive and has a short serum half-life, increasing even in moderate muscular damages and returning rapidly to normality [201]. For that reason, only large increases in serum CK activity are considered to have clinical significance, particularly if combined with the analysis of the less specific but longer-lasting AST [223]. Although CK and AST are considered more sensitive indicators of muscular damage than LDH and ALT [186,188], it is wise to analyse the activity of all four enzymes to account for interspecific variability in sensitivity and response to stressful stimuli.

Although all the clinical, haematological, and serum biochemical variables described above are influenced by the stress response and the pathophysiological effects of capture stress and capture myopathy, each can also be affected by other physiological processes or pathological conditions. Consequently, the assessment of any single variable provides limited and nonspecific information regarding the pathophysiological status of the animal. In contrast, integrated monitoring of the proposed panel of variables offers a reliable, sensitive, and specific assessment of the physiological status of captured and handled free-ranging wild ungulates. Continuing research on wild ungulate capture stress assessment is generating new indicators of animal welfare and health, thereby providing a more comprehensive and accurate understanding of the pathophysiological status of captured individuals, improving prognostic assessment, and enabling the development of more refined tools for evaluating capture and handling protocols. Thus, skeletal muscle intracellular molecules such as anserine and 3-methyl-L-histidine have been proposed as new indicators of muscular damage [94]. Even more useful, the determination of cardiac troponins has been proposed and is gaining relevance as a potential indicator disentangling heart muscle and striated muscle damages, thus providing differential diagnostic and prognostic value for each organ [224,225,226], particularly if combined with the determination of CK, AST, ALT, and LDH [170,171]. However, increasing the number of samples to be collected and analyses to be performed on the field introduces additional complexity and may suppose a longer time of restraint and handling of the animal, increasing the risk for pathological consequences. Thus, improvements in the monitoring protocol through data recording, sampling, and analyses must be balanced against the overall capture and handling protocol.

The determination of these clinical, haematological, and serum biochemical variables altogether can be repeated over time during post-capture restraint to further allow quite a complete characterisation of the intensity of the challenge posed to homeostasis by physiological stress, as well as the welfare, health, and death hazard caused by the capture and handling protocol. However, not all these variables can be assessed in real time and in situ during the capture operation. What is their usefulness, then?

**Figure 4:**
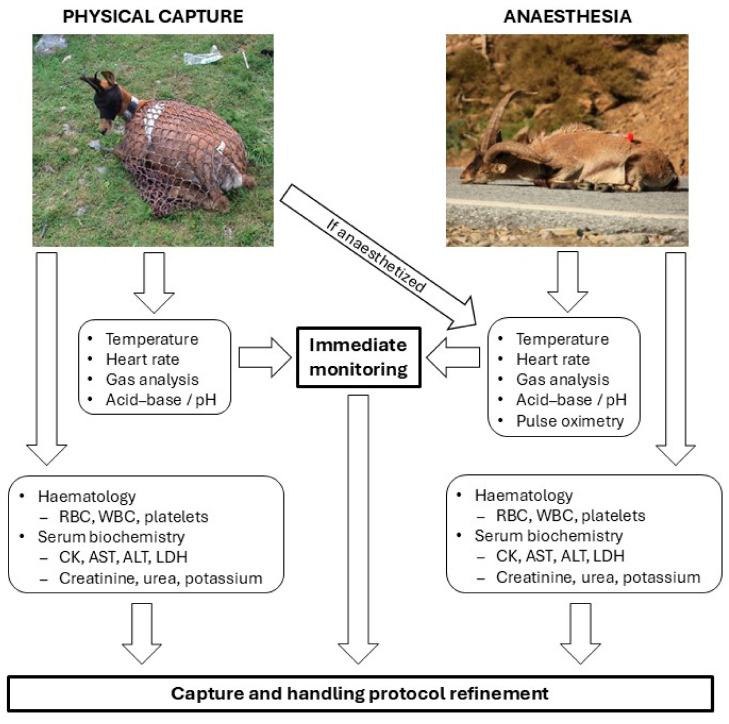
Monitoring and analytical opportunities and applications in physically captured, anaesthetized, and physically captured and then anaesthetized European wild ungulates.

While the methods, stress response, and pathophysiological changes experienced by physically and chemically captured wild ungulates have been widely reported (references in Table 1) [170,171,174,175,176,177], protocols to assess their welfare and health, as well as decision thresholds for care intervention or release, are limited and not well-established. The possibilities to measure, assess, and monitor stress in captured wild ungulate are conditioned by the capture and handling protocol and may serve to a dual objective, namely (1) immediate monitoring and knowledge of the pathophysiological status of the captured individual; and (2) identifying the pathophysiological compromise posed by the capture and handling protocol, so the data and information can be used to refine the protocol, thus improving animal welfare and health in subsequent captures (Figure 3 and Figure 4).

The monitoring possibilities for each capture protocol (physical capture, anaesthesia, and physical capture followed by anaesthesia) and their usefulness (immediate and/or to refine capture and handling protocol) are summarised in Figure 4. Not all the procedures in Figure 4 are to be used in every single capture protocol, but it should be considered as a dynamic guideline where new variables (such as specific markers of muscular or heart cell damage [94,224,225,226]) can be added and others may eventually be eliminated. While immediate monitoring of clinical variables is a usual practice of professionals capturing and handling free-ranging European wild ungulates, detailed data recording and sample collection for posterior analyses is less frequent, mostly performed by researchers. Finally, using the information obtained from both immediate clinical monitoring and posterior analyses to critically revise, improve, and refine protocols is scarcely carried out. Few if any reports on capture and handling protocol improvement after such assessment exist for free-ranging European wild ungulates. The responsibility of researchers, managers, stakeholders, and anybody capturing and handling wildlife is not only to ensure animal welfare, health, and survival during capture and manipulation; they should also use the available tools to record all the information and collect and analyse all the samples necessary to assess, improve, and refine the capture and handing protocol.

**Figure 5:**
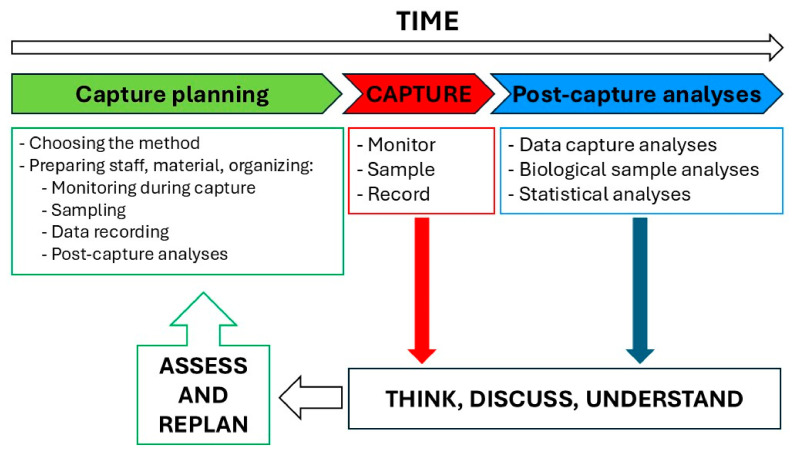
Proposed flow chart for the planning, development, monitoring, assessment, evaluation, and modification of protocols to capture and handle European wild ungulates.

Thus, capture and handling protocols should be designed and implemented including not only monitoring but also data recording, sample collection, and post-capture data and sample analyses (Figure 5). Protocols should completely describe in detail, at least:

1. Capture methodology, including not only capture method but also all the procedures to be carried out.
2. Post-capture handling, including transport if it is the case, and release.
3. Monitoring of variables to serve both immediate individual assessment and protocol revision and improvement.
4. Data recording with the same purposes as above.
5. Sample collection and processing.
6. Data and sample analyses.
7. Use of the results to revise and refine the capture and handling protocol.

The information obtained from the complete capture, handling, monitoring, recording, sampling, and analysis protocol should be used for revision of the protocol itself to adapt to the specific species, environment, objectives, conditions, and circumstances. The protocols should be sufficiently adaptable and flexible to accommodate new procedures while eliminating unnecessary, time consuming, or potentially harmful practices. Protocols should be reviewed and revised regularly in the light of findings obtained from data and sample analyses to ensure the continual alignment of capture, handling, and stress assessment practices with state-of-the-art methodologies and techniques, thereby maximising animal welfare, health, and ultimately survival.

Finally, although beyond the scope of this review, the information gathered through such protocols should be assessed against the results of post-capture monitoring of the released animals, since reduced pathophysiological effects of capture and handling should correspond with improved conditions and survival after release. This should also allow to further investigate the consequences of stress-related capture myopathy and relate them to the changes observed through real-time monitoring, data collection, and sample analyses.

## 6. Conclusions

The concern and responsibility when capturing wildlife about animal welfare, health, and survival, both during capture and handling and after release, requires not only individual monitoring, but also to gain knowledge on the pathophysiological challenge caused, thus allowing for the revision and refinement of protocols. Available tools to assess not only traumatic injuries and mortalities, but also the capture stress induced, should be incorporated into protocols to capture, handle, monitor, record, sample, and analyse wild ungulates.

Since single markers would not be sensitive and specific enough to capture the physiological status of the captured and restrained wild ungulates, a panel simultaneously evaluating the intensity and duration of the perceived alarm (temperature, heart rate, pulse oximetry, blood gas, and erythrocyte, leucocyte, and platelet counts), and muscle damage and renal compromise (serum CK, AST, ALT, and LDH activities and creatinine, urea, and potassium concentrations) is recommended. Moreover, repeated sampling over time should be carried out in long post-capture protocols, to monitor the dynamics and evolution of the challenge posed to homeostasis. The development, validation, and standardisation of new minimally invasive markers of physiological status not affected by sampling or management to be measured is required to advance in the monitoring of capture and handling stress.

The information obtained should serve to constantly revise, adapt, and improve these protocols for performance and animal welfare, health, and survival. While this review focuses on free-ranging wild European ungulates, it may serve as a basis for the refinement of capture, handling, and stress assessment protocols for ungulates in other regions or for other taxa, with the adequate adaptions. The generated knowledge should eventually lead to the creation and development of specific guidelines for stress, welfare, health, and survival assessment for the capture, handling, and post-release follow-up of wild ungulates, including not only lesions but also capture stress.

## Figures and Tables

**Table 1:** Methods used to capture wild ungulates in Europe.

Species	Method	References
*Alces alces* (moose)	Teleanaesthesia	[59,60,61,62,63,64,65,66,67]
*Bison bonasus* (European bison)	Restriction crate	[68]
	Teleanaesthesia	[69,70,71,72,73]
*Capra ibex* (Alpine ibex)	Box-trap	[53,74,75,76]
	Drop-net	[74]
	Snares	[76]
	Teleanaesthesia	[2,53,74,75,76]
*Capra pyrenaica* (Iberian ibex)	Box-trap	[77,78]
	Box-trap + anaesthesia	[55]
	Corral-trap	[6]
	Drive-net	[5,77,79,80]
	Teleanaesthesia	[81]
*Capreolus capreolus* (roe deer)	Box-trap	[29,82,83,84,85]
	Drive-net	[5,82,86,87,88,89,90]
	Mesh-trap	[91]
	Net-traps	[82,83,84]
	Teleanaesthesia	[92,93]
*Cervus elaphus* (red deer)	Box-trap	[94]
	Drive-net	[54]
	Drop-net	[32]
	Teleanaesthesia	[54,94,95,96,97,98,99,100]
*Dama dama* (fallow deer)	Restriction crate	[101]
	Teleanaesthesia	[101,102,103,104,105,106,107]
*Ovibos moschatus* (muskox)	Teleanaesthesia	[108,109,110,111]
*Ovis aries musimon* (European mouflon)	Corral-trap	[112,113,114]
	Snares	[115]
	Teleanaesthesia	[116,117]
*Rangifer tarandus* (reindeer)	Corral-trap	[118]
	Drive-net	[119]
*Rupicapra pyrenaica* (southern chamois)	Box-trap + anaesthesia	[120]
	Drive-net	[5,121,122,123,124,125]
	Snares	[126,127]
	Teleanaesthesia	[128]
	Up-net + anaesthesia	[120]
*Rupicapra rupicapra* (northern chamois)	Snares	[45]
	Teleanaesthesia	[56,116,129,130,131]
	Up-net	[30]
*Sus scrofa* (wild boar)	Box-trap + anaesthesia	[1,21,23,132,133]
	Corral-trap	[134,135]
	Corral-trap + anaesthesia	[1,23]
	Drop-net + anaesthesia	[20,21,23]
	Teleanaesthesia	[21,23,100]

## Data Availability

No new data were created or analyzed in this study. Data sharing is not applicable to this article.
